# Covalent fragment inhibits intramembrane proteolysis

**DOI:** 10.3389/fmolb.2022.958399

**Published:** 2022-09-07

**Authors:** Angela Eden, Jing Zhao, Yuanyuan Xiao, James Gibson, Chunyu Wang

**Affiliations:** ^1^ Center for Biotechnology and Interdisciplinary Studies, Troy, NY, United States; ^2^ Department of Chemistry and Chemical Biology, Troy, NY, United States; ^3^ Department of Biological Sciences, Troy, NY, United States

**Keywords:** Alzheimer’s disease (AD), targeted covalent inhibitor, covalent warhead, amyloid precursor protein (APP), intramembrane proteolysis

## Abstract

Alzheimer’s disease (AD) is a serious public health crisis with only one current modifying treatment. The reduction of amyloid load by targeting γ-secretase (GS) has been a leading approach in AD drug discovery and development. Despite the focus on GS inhibition, multiple GS inhibitors (GSIs) have failed in clinical trials as a result of side effects including exacerbated cognitive decline. These side effects are largely attributable to inhibition of normal GS function. Standard enzyme inhibitors target catalytic or allosteric sites of the enzyme, including the active site presenilin, as previous GSIs did. To avoid issues observed from broad-spectrum GSIs we discovered that fragment 6H8 that covalently binds to the substrate of GS, the transmembrane domain of amyloid precursor protein (APPTM). Nuclear Magnetic Resonance (NMR) Spectroscopy combined with MALDI-TOF-MS established 6H8 covalently binds to APPTM. 6H8 acts as a Michael acceptor and covalently links to the side chain amines of lysine residues, specifically targeting a cluster of C-terminal lysines K53–K55. Through this modification, 6H8 can inhibit intramembrane proteolysis of an archaeal homolog of presenilin (the active subunit of GS) *via* substrate binding with a 2–4 μM IC_50,_ determined by a gel-based cleavage assay. 6H8, while too small to be an effective drug candidate, can be combined with a specific non-covalent partner and function as an effective covalent warhead of a targeted covalent inhibitor (TCI). The future development of the 6H8 fragment into the covalent warhead of a TCI is, to our knowledge, a novel approach to AD drug discovery.

## Introduction

Alzheimer’s Disease (AD) is a neurological disease currently affecting close to six million Americans, projected to rise dramatically over the next 10 years (Cummings and Cotman, 1995; Alzheimer’s Association, 2022). AD is the sixth leading cause of death overall and the fifth for people over the age of 65. With our rapidly aging population, a cure for AD remains at the forefront of the unmet medical needs of this nation (Cummings and Cotman, 1995; Alzheimer’s Association, 2022). A major neuropathological hallmark of AD is the presence of senile plaques in the cerebral cortex and hippocampus (Price et al., 1998). Senile plaques, also called amyloid plaques, are mainly composed of extracellular aggregates of amyloid β-peptides (Aβs); and it has been hypothesized that these plaques initiate a pathological cascade resulting in cognitive decline (Selkoe, 2001; Selkoe and Hardy, 2016).

γ-secretase (GS) has four essential proteins subunits, presenilin (PS), nicastrin (Nct), anterior pharynx-defective 1 (Aph-1), and presenilin enhancer 2 (Pen-2), (Esler et al., 2000; Li Y. et al., 2000; Li Y. M. et al., 2000; De Strooper, 2003; Wolfe and Kopan, 2004; Wolfe, 2006; Ahn et al., 2010) where PS is the catalytic subunit (Jarrett et al., 1993; Francis et al., 2002; Haapasalo and Kovacs, 2011; Coric et al., 2012). GS is an intramembrane-cleaving protease (I-CLiP) that hydrolyses peptide bonds located inside the membrane lipid bilayer allowing for the release of bioactive peptide fragments (Haze et al., 1999; Niwa et al., 1999; Brown et al., 2000; Lal and Caplan, 2011; Lichtenthaler et al., 2011). I-CLiPs are critical for a myriad of biological processes and therefore are implicated in a number of disease pathologies like AD (Winter-Vann and Casey, 2005; Lichtenthaler et al., 2011; Düsterhöft et al., 2017). While there are multiple classifications of I-CLiPs, GS (and subsequently PS) is a di-aspartyl protease which is characterized by two catalytic aspartates. One catalytic aspartate is adjoined by a GXGD motif where G is glycine, X is any amino acid, and D is aspartate (Steiner et al., 2000; Fluhrer et al., 2009). GS cleaves APP’s transmembrane domain, in addition to over 90 other physiological substrates including Notch (Francis et al., 2002; Haass and Steiner, 2002; Haapasalo and Kovacs, 2011). As a result, inhibiting GS may result in major disruptions of homeostatic functions, such as cell adhesion and signaling.

**FIGURE 1:**
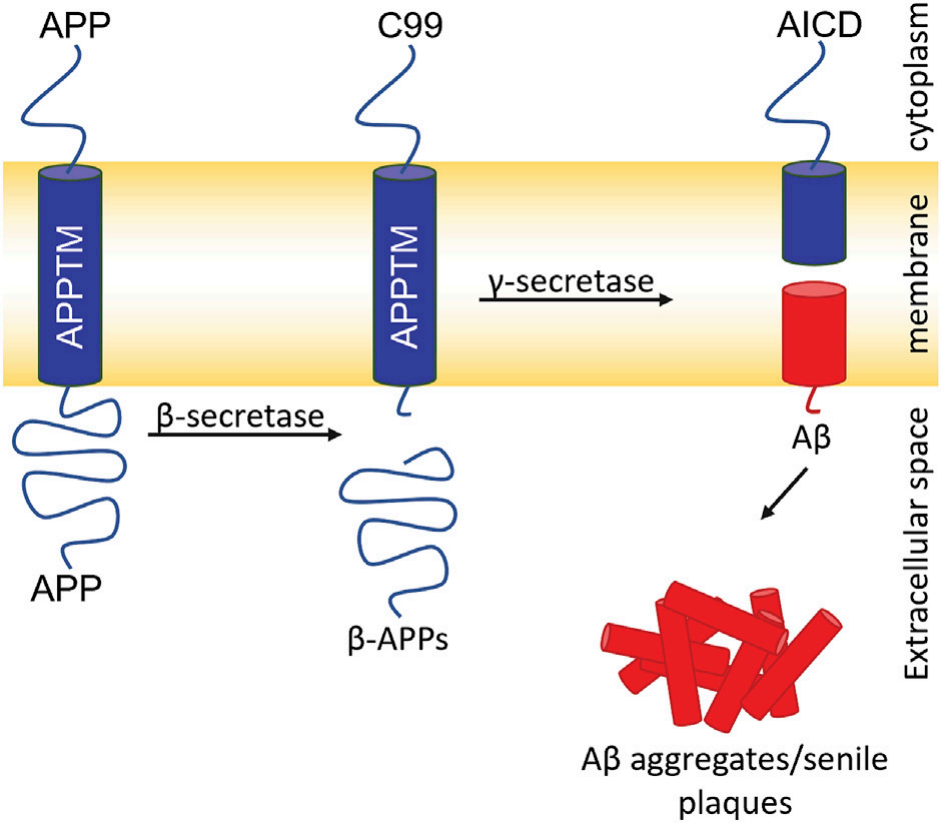
Amyloidogenic pathway for APP processing and Aβ generation. Cleavage of APP by β-secretase generates the 99 C-terminal residues of APP (C99). Intramembrane proteolysis of C99 by GS generates Aβ40/42. Aβ aggregates generating senile plaques a major pathogenic hallmark of AD.

Aβ, the major component of senile plaques, is generated from amyloid precursor protein (APP). APP is cleaved consecutively by two proteases: β- and γ-secretase (Figure 1) (Jarrett et al., 1993). β-secretase cleaves APP and generates a C-terminal 99 residue construct (C99). GS then cleaves C99 in the transmembrane domain (APPTM) which releases Aβ into the extracellular or luminal space (Jarrett et al., 1993). Aβ has two major isoforms: Aβ40 and Aβ42, composed of 40 and 42 residues, respectively. Aβ40 is benign in comparison to Aβ42, which has a much higher propensity to aggregate into neurotoxic oligomers and fibrils.

Despite the focus on Aβ as a potential drug target for AD, all but one (aducanumab) anti-Aβ drug failed in clinical trials (Coric et al., 2012; Doody et al., 2013; Coric et al., 2015; Ferrero et al., 2016; Abyadeh et al., 2021). Two broad-spectrum GS inhibitors (GSIs), avagacestat and semagacestat, failed due to worsening cognition in patients in addition to other serious adverse effects (Coric et al., 2012; Doody et al., 2013; Coric et al., 2015). As of now, the only FDA-approved anti-Aβ treatment is aducanumab, which is not without controversy. The basis of the controversy is insufficient data demonstrating the efficacy of the drug in improving the cognitive function of AD patients (Ferrero et al., 2016; Abyadeh et al., 2021). Despite the failure of GSIs in clinical trials and the aducanumab controversy, there is compelling evidence that Aβ is a causative agent in AD, including human genetics of familial AD (FAD) (Selkoe, 2001; De Jonghe et al., 2002; Chen et al., 2014) and Down’s syndrome, (Lejeune et al., 1959; Head et al., 2012), Aβ toxicity and related neuron inflammation, (Eng et al., 2004; Shankar et al., 2008), and potentiation of tau pathology (Jin et al., 2011).

Familial AD (FAD) is a genetic form of AD characterized by early-onset dementia. FAD is caused by mutations within the APP/GS cascade. Most of the FAD mutations occur within PS genes highlighting the role of GS in AD pathology (Haass and Steiner, 2002). One feature of FAD is an increased Aβ42/Aβ40 ratio which contributes to the early onset of AD. To better characterize AD and FAD, our lab has previously solved the structure of APPTM, the substrate of GS for Aβ generation, *via* solution NMR in micelles (Chen et al., 2014). We also studied FAD mutations (V44M and V44A) within APPTM and found that these mutations likely enhance the flexibility and therefore the accessibility of the initial Ɛ-cleavage site for the Aβ42 production line, contributing to the higher Aβ42/Aβ40 ratio seen in FAD (Fluhrer et al., 2009). Our lab also demonstrated that the C-terminal lysine cluster of APPTM (K53-K55) participates in the initial docking of APPTM to intramembrane protease GS, coupled with helical unwinding to ready the substrate for peptide bond hydrolysis (Clemente et al., 2018). Recently, the cryo-EM structure of GS complexed with APP substrate revealed an α-helical to β-strand transition at the C-terminus of APPTM. This transition exposes the initial ε-cleavage sites to interact with the active site of γ-secretase (Zhou et al., 2019).

The large number of endogenous substrates of GS presents a significant obstacle to the development of GSIs, exemplified by the aforementioned clinical trial failures (Haapasalo and Kovacs, 2011). These trials were ultimately discontinued due to serious side effects, attributed to the suppression of γ-secretase activity with other endogenous substrates such as tyrosinase, Notch, and N-cadherin (De Jonghe et al., 2002; Marambaud et al., 2003; Crump et al., 2012; Doody et al., 2013). Additionally, GSIs that bind the catalytic subunit of GS (PS) pose a particular issue; PS is involved in learning, memory, and neuronal survival, which may have contributed to the exacerbated cognitive decline observed in clinical trials (Saura et al., 2004; Wines-Samuelson et al., 2010; Haapasalo and Kovacs, 2011; Watanabe et al., 2012; Barthet et al., 2013). Considering that APPTM is the substrate for GS that generates Aβ, an alternative approach previous explored by the Wang lab is to target the substrate (APPTM) to reduce the production of Aβ (Zhao et al., 2020). A substrate-specific inhibitor is not anticipated to affect GS complex formation, presenilin function, and most importantly normal function of GS with other endogenous substrates, thereby reducing the potential side effects of broad GSIs.

The main consideration for making APPTM the target of drug discovery is that transmembrane helices, like APPTM, are difficult drug targets, due to the lack of binding pockets. This issue can be mitigated with the implementation of a covalent modifying drug compound that does not require a specific binding pocket to dock while also benefiting from the zero off rate. We report a novel compound, 6H8, which covalently modifies APPTM at three adjacent lysines in the C-terminal juxtamembrane. With only one disease-modifying therapy approved by the FDA, 6H8 serves as a covalent warhead poised to be used in rational drug discovery that spares the activity of GS and as a novel drug discovery avenue (Ferrero et al., 2016; Abyadeh et al., 2021).

## Results

### 6H8 binds the C-terminus of transmembrane domain of amyloid precursor protein as shown by 2D NMR

**FIGURE 2:**
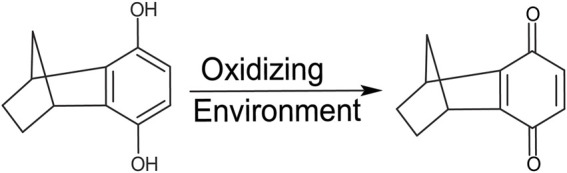
Structure of 6H8. The hydroquinone moiety is readily oxidized to its active quinone form.

**FIGURE 3:**
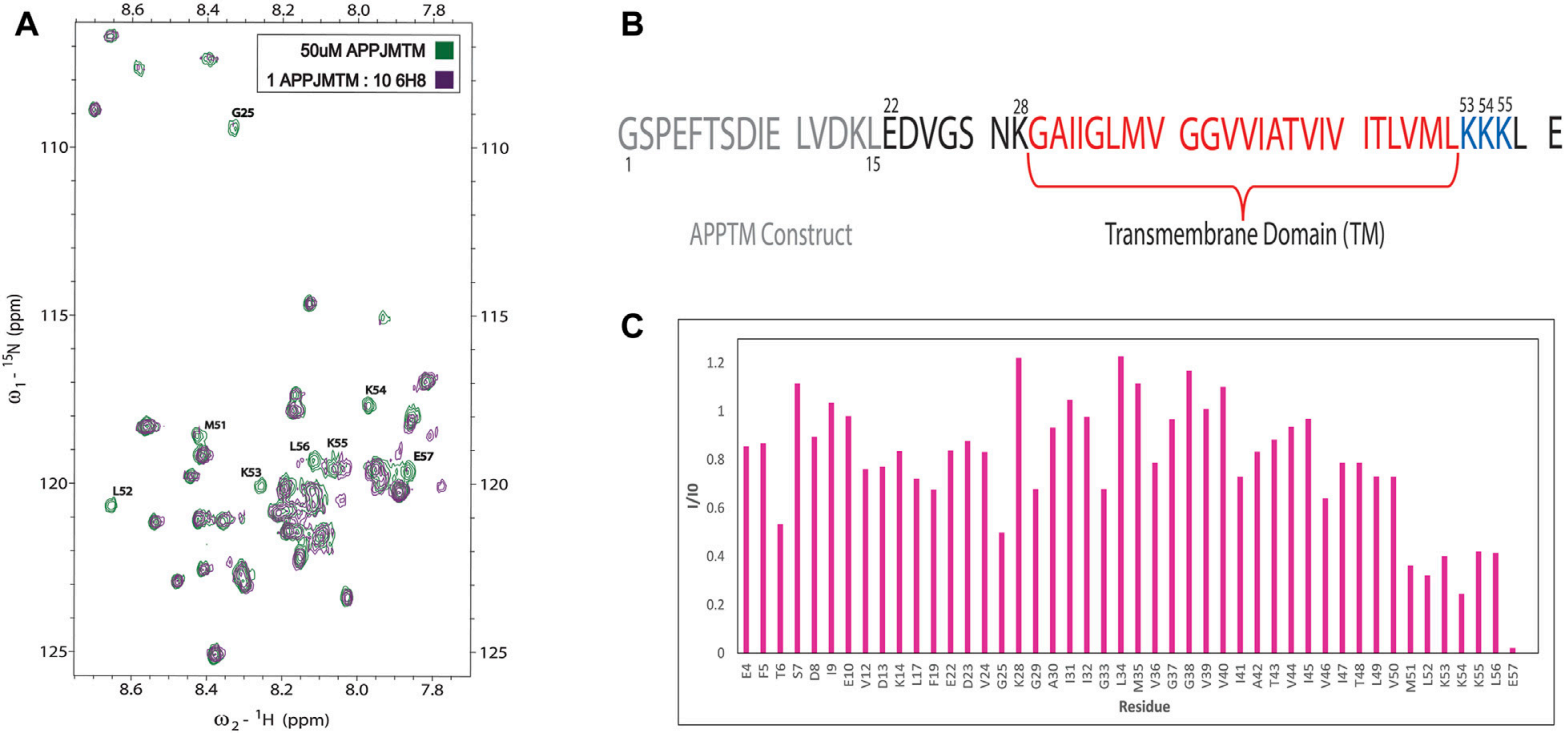
NMR of titration of APPTM with covalent fragment 6H8. **(A)**n overlay of 2D ^1^H-^15^N TROSY spectrum of APPTM with (green) and without 6H8 (purple). Resonances with the largest changes in peak intensity were labeled by residue type and number. **(B)** Sequence of APPTM construct used in all experiments. Residue numbering switches to Aβ numbering at residue E22 **(C).** Selective line broadening observed in 3A is quantified *via* I/I_0_ calculation, where the peak height at each residue of the APO spectrum (green) is I_0_ and the peak height per residue of titration point (purple) is (I). Substantial line broadening is observed at and near the C-terminal triple Lys motif indicated by a low (under 0.5) I/I_0_ value.

Initial NMR screening of a fragment library (Maybridge Ro3 1000 library) using APPTM as the target yielded 6H8 as a binder of APPTM. We then explored the binding interaction using 2D ^1^H-^15^N transverse relaxation optimized spectroscopy (TROSY) (Figure 3A), which correlates the proton and nitrogen of the amide group of each individual residue in the sequence of APPTM (Figure 2). To have the optimal line width of APPTM in dodecyl phosphocholine (DPC) micelles, we used a TROSY sequence for the 2D NMR experiments, which yields a fingerprint-like spectrum that can be used to identify and monitor changes in the protein. Binding events and changes to the local chemical environment of the protein can be observed with a selective line broadening indicated by peak height reduction into the baseline at or near that residues’ side.

Overall, 6H8 had a considerable reduction in signal at and near Lys residues. Figures 3A,C highlight specific residues of APPTM impacted by the 10-M excess of 6H8 after 3.5 h of incubation at 37°C. Peaks that experience height reduction, indicated by an I/I_0_ value under 0.5, where over half the initial signal is lost, undergo the greatest chemical change with the addition of 6H8. Peaks with the lowest I/I_0_ values were at the C-terminal lysine residues K53, K54, and K55 (I/I_0_ values: 0.40, 0.25, and 0.42 respectively) and their neighbors M51, L52, L56, and E57 (I/I_0_ values: 0.36, 0.32, 0.41, and 0.22 respectively). In analyzing these values and overall spectra, we observe the binding interaction between 6H8 and APPTM specifically targets the C-terminal lysine cluster given the reactivity of the free amine groups at the end of lysine sidechains. With clear interaction between 6H8 and APPTM confirmed by 2D TROSY NMR, we wanted to further explore if 6H8 could inhibit the intramembrane proteolysis of APPTM in the amyloidogenic cascade (Figure 1).

### 6H8 inhibits cleavage by presenilin homologue MAMRE50

**FIGURE 4:**
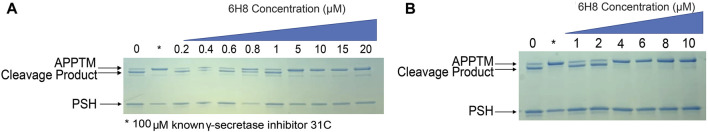
6H8-modified substrate inhibits the cleavage of APPTM by PSH MAMRE50 (PSH) in a gel-based assay. Varying concentrations of 6H8 were incubated with APPTM and then removed before assay to eliminate interference with PSH. Covalently modified APPTM was further incubated with PSH at 37°C. The extent of cleavage was determined *via* SDS-PAGE gel electrophoresis. **(A)** Initial screening of 6H8 in this assay was done from 0.2 to 20 μM. IC_50_ was under 10 μM. **(B)** Secondary assay with a smaller range of 6H8 (1–10 μM) was performed; the IC_50_ was between 2 and 4 μM.

To determine if 6H8 could inhibit APPTM cleavage by PSH, an *in vitro* gel-based cleavage assay was employed using an archaeal homolog of presenilin (PSH) MAMRE50. This homolog replicates most structural and biochemical features of presenilin/GS, serving as a substitute for fully formed GS, which is difficult to overexpress and purify (De Strooper et al., 1998; Li Y. M. et al., 2000; Selkoe and Wolfe, 2007; Li et al., 2012; Dang et al., 2015; Zhao et al., 2020). APPTM was incubated with PSH for 24 h at 37°C to obtain full cleavage of APPTM under these conditions. The extent of cleavage was monitored by the intensity of the lower molecular weight band that appears slightly below the fully intact APPTM, indicating successful intermembrane proteolysis of APPTM by PSH (Figure 4). (Li et al., 2012; Dang et al., 2015)

Control samples were run with every gel for both positive and negative hits. Our negative control is a sample containing only APPTM and PSH allowing for full uninhibited cleavage; whereas our positive control utilizes a known PSH inhibitor at 31°C to represent fully inhibited cleavage (Zhao et al., 2006). 6H8-modified APPTM was incubated with PSH to determine the extent of cleavage at various 6H8 concentrations (Figure 4A). The dose-dependent reduction in the intensity of the cleavage band on the gel indicates considerable inhibition of PSH cleavage of 6H8-modified APPTM. Initial IC_50_ screening was performed with 6H8 concentrations between 0.2 and 20 μM (Figure 4A) showing cleavage inhibition between 0.8 and 5.0 μM. Following the initial screen, a smaller range of 6H8 concentrations was employed for a more precise IC_50_ determination. The smaller 6H8 range was between 1 and 10 μM, with complete inhibition observed between 2 and 4 μM. Given the low, micromolar IC_50,_ further experiments were conducted with 6H8 for future drug development.

### 6H8 covalently modifies transmembrane domain of amyloid precursor protein

**FIGURE 5:**
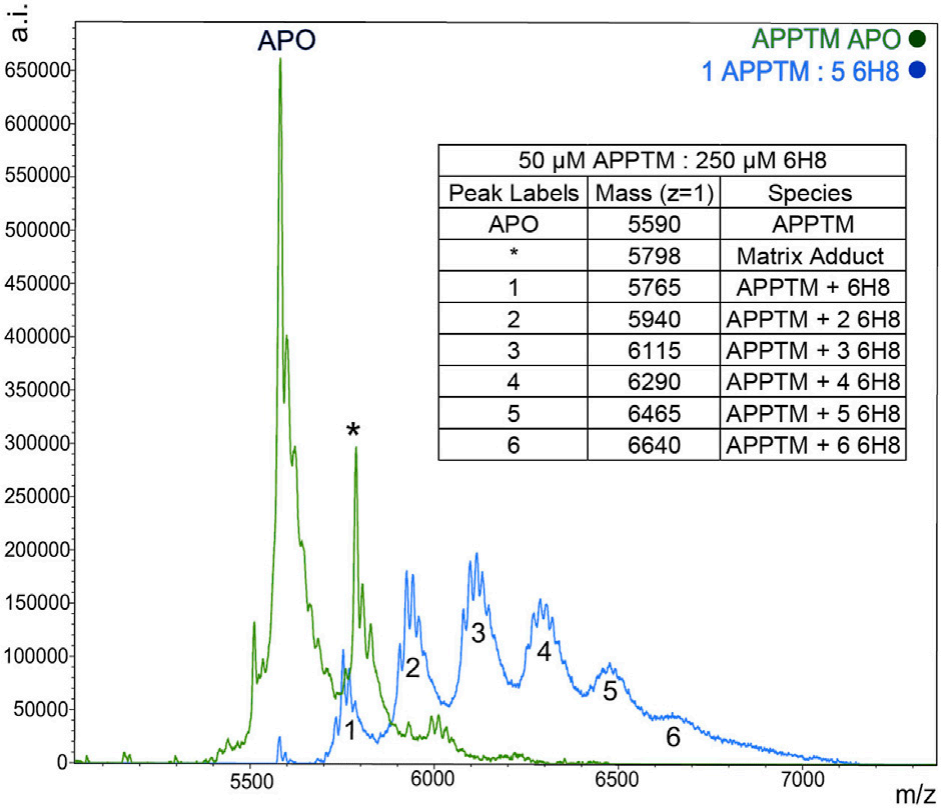
MALDI-TOF MS demonstrates a covalent modification of APPTM by 6H8. Multiple additions of 6H8 are exhibited in peaks one to five of the blue spectrum. Peak one at 5,598 $\frac{m}{z}$ corresponds to the addition of active 6H8 minus one hydrogen ( $\Delta \frac{m}{z}\;175$ ). The following peaks two to five correspond to multiple additions of active 6H8 molecules (Δ $\frac{m}{z}\;$ = 350, 525, 700, 875, and 1,050, respectively).

MALDI-TOF-MS was utilized to further characterize the binding interactions between 6H8 and APPTM. NMR can illuminate many things about the binding interactions, but it lacks the ability to distinguish between covalent and non-covalent interactions. Covalent modification is advantageous for drug targets like APPTM due to its lack of binding pocket as a helical dimer. Incubation of APPTM in the presence of 5 M excess of 6H8 shows complete conversion of the APO protein to covalently modified APPTM with up to six additions of 6H8 (Figure 5). With multiple Lys in the sequence (Figure 3B), there are ample positions for multiple additions of 6H8 observed in the MALDI-TOF spectrum (Figure 5). Differences in the original mass of the APO peak (5,590 m/z) correspond to the molecular weight of 6H8 (176 Da) missing lacking a hydrogen ( $\Delta \frac{m}{z}\;175$ ) being added to APPTM. Molecular weight changes corresponding to multiple additions can be seen in Figure 5, with a maximum of six additions, shifting the APO protein mass by 1,050 $\frac{m}{z}$ . Confirmation of covalent modification provides valuable information when determining the structure-activity relationships as we know there must be covalently binding moiety present under given oxidizing conditions.

### Reducing conditions eliminate 6H8 binding to transmembrane domain of amyloid precursor protein and allows for complete cleavage by presenilin homologue

In standard reaction/oxidizing conditions (25 mM HNa_2_PO_4_, 4% DPC, pH 7.2), 6H8 readily binds to APPTM. Given that the hydroquinone moiety is known to readily oxidize into quinone, and the time-dependent color change of 6H8 stocks in an aqueous buffer (from colorless to yellow-brown), we hypothesized that oxidation causes 6H8 to act as a pro-drug-like molecule (Radel et al., 1982; Yadav et al., 2008). The quinone conversion reveals the required *α*, *β* unsaturated ketone for a Michael addition with the free amines of Lys side chains and the N-terminus.

**FIGURE 6:**
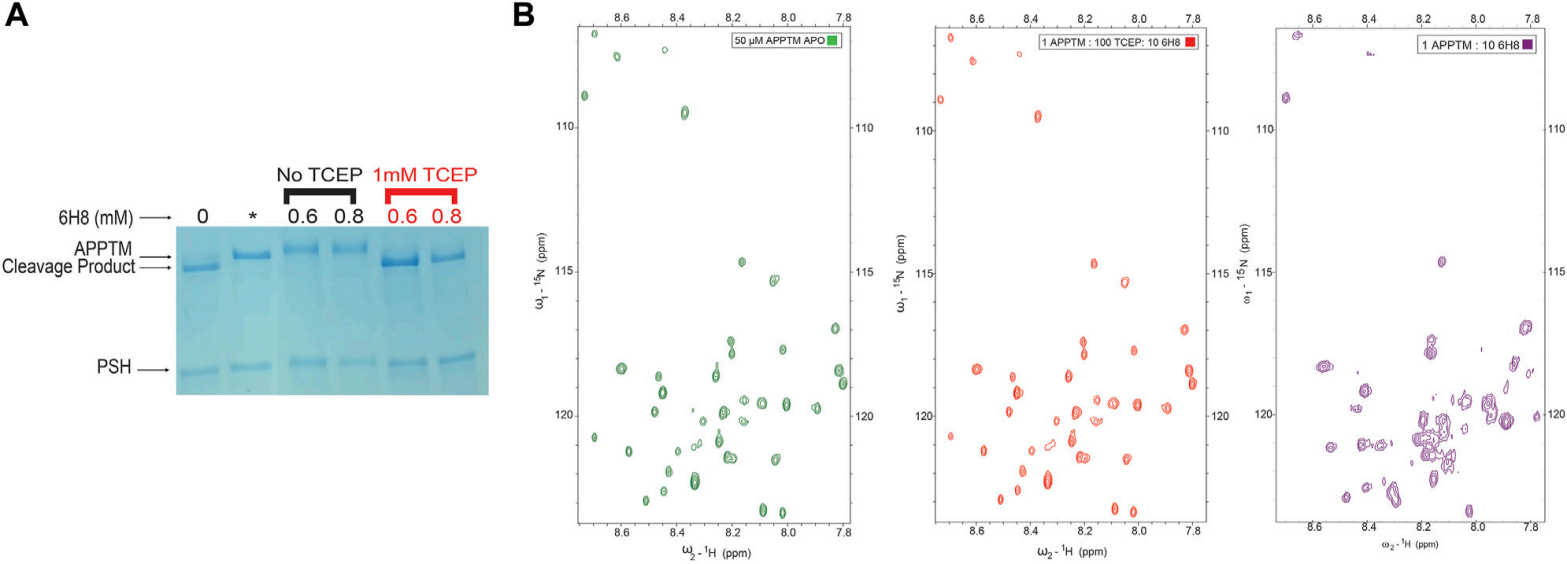
Efficacy of 6H8 in inhibiting intramembrane proteolysis and binding to APPTM under reducing conditions **(A)**. To confirm that efficacy of 6H8 can be eliminated with reducing agent another PSH gel assay was run with excess 6H8. With an excess of 0.6 and 0.8 mM 6H8 with and without TCEP (black and red respectively) as the reducing agent 6H8’s efficacy is eliminated **(B).** Comparison of 2D 1H-15N TROSY spectrum of 50 μM APPTM APO (green) and 1:100:10 APPTM: TCEP: 6H8 (red). Notably K53-K55 are unaffected by 6H8 in the presence of TCEP indicating that in the presence of excess reducing agents the binding of 6H8 is eliminated. Final spectrum is 1:10 APPTM:6H8 (purple). There is a significant difference between this spectrum and its neighboring red spectra that has excess TCEP along with 6H8, indicating elimination of reactivity of 6H8 under reducing conditions compared to oxidizing conditions.

To test this hypothesis, we introduced the reducing agent tris (2-carboxyethyl) phosphine (TCEP) to prevent the conversion of 6H8 from inactive hydroquinone to active quinone form. Initial testing of this hypothesis was done using the PSH cleavage assay. An excess of 6H8 (0.6 and 0.8 mM) was incubated with APPTM and PSH both with and without 1 mM of TCEP (Figure 6A, red and black respectively). Given that the IC_50_ of 6H8 was between 2 and 4 μM as determined by this PSH assay, this assay was done with an extreme excess of 6H8. Under normal oxidizing conditions, both 0.6 and 0.8 mM 6H8 completely inhibit PSH cleavage of APPTM as no cleavage product is present. In contrast, the lanes with the same excess amounts of 6H8 incubated with 1 mM of reducing agent TCEP demonstrate complete cleavage of APPTM (Figure 6). These results confirm our hypothesis that 6H8 needs to be in an oxidizing environment to inhibit cleavage by PSH and by extension GS.

### 6H8 binding to the C-terminus of transmembrane domain of amyloid precursor protein is diminished in reducing conditions as shown by 2D NMR

As a follow-up to the PSH inhibition assay, a 2D NMR TROSY experiment was performed to determine if a reducing agent can inhibit binding to APPTM in its entirety. Figure 6B exemplifies a 2D NMR TROSY under the same conditions as the TROSY depicted in Figure 3A (25 mM Na_2_PO_4_, 4% DPC, 90% H_2_O/10% D_2_O) save for the presence of ten-fold molar excess of TCEP in relation to 6H8. In the presence of TCEP, there is no binding between 6H8 and APPTM, as demonstrated by the near-identical spectra between apo APPTM and APPTM with 6H8 in reducing conditions (APPTM 50 μM: TCEP 5000 µM: 500 µM 6H8). We provide a comparison with an APPTM incubated with 10-fold molar 6H8 in oxidizing conditions to demonstrate the significant difference with active APPTM-6H8 binding (1:10 APPTM: 6H8; Figure 6B, right-most spectrum). These results affirm that the oxidizing environment is essential for the covalent linkage between 6H8 and APPTM.

### 6H8 covalently binds transmembrane domain of amyloid precursor protein under oxidizing conditions *via* a michael addition mechanism

**FIGURE 7:**
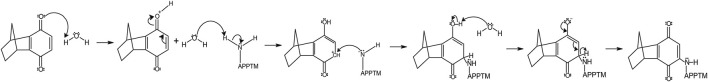
The proposed mechanism of covalent modification of APPTM by 6H8 at Lys residues follows a Micheal addition mechanistic scheme.

We propose that the reaction between 6H8 and APPTM follows a water-catalyzed Michael addition similar to that reported by Yadav et al. (2008). The first step involves oxidization of the hydroquinone moiety of 6H8 to a quinone structure with water as the catalyst. This quinone structure provides *α*, *β* unsaturated ketone (Michael acceptor) for Michael addition. The amine group at the end of lysine side chains K53-K55 (Michael donor) performs a nucleophilic attack on the unsaturated ketone, followed by solvent-driven proton abstraction and reestablishment of resonance (Figure 7). Water is the most likely catalyst in our system as an aqueous buffer (25 mM Na_2_PO_4_, 4% DPC, pH 7.2). Figure 7 illustrates the mechanism described above using water as the catalyst for the reaction at near-neutral pH (pH 7.2).

## Discussion

Developing disease-modifying strategies against AD is crucial as the US population ages in the coming decades. Combating senile plaque formation is a potential drug target to slow down the progression of AD. Senile plaques are composed of aggregated Aβ which are generated during the intermembrane proteolysis of APPTM in C99 (Barthet et al., 2013). Therefore, targeting the C99/γ-secretase interface is a legitimate approach to reducing Aβ generation and overall amyloid load. Previous clinical trials targeting the amyloidogenic pathway-focused efforts on inhibition of γ-secretase (GS), however, these trials were discontinued due to acute side effects and accelerated cognitive decline. Serious side effects likely stemmed from inhibition of cleavage of the 90 other known physiological substrates of GS. The inhibition of presenilin likely resulted in the worsening cognition because of its established role in neuronal survival, learning, and memory retention (Saura et al., 2004; Wines-Samuelson et al., 2010; Crump et al., 2012; Watanabe et al., 2012). To avoid the pitfalls of previous trials, we focused our efforts on compounds that target the amyloid substrate of GS. This approach would allow GS to maintain normal function for other substrates as well as avoid inhibiting the critical functions of presenilin.

Utilizing NMR, MS, and enzyme cleavage assays, we report the discovery of a novel fragment named 6H8 that covalently binds APPTM and inhibits cleavage of PSH, a homolog of the catalytic subunit of GS. The discovery of this compound follows our discovery of a similar compound C1 that was the first reported covalent GS inhibitor (Barthet et al., 2013). The binding interaction of 6H8 to APPTM is similar to C1 in both mechanism and site of binding, undergoing Michael additions with the free amines at K28 and K53-55 (Takami et al., 2009).

NMR titration showed a significant interaction of 6H8 to the C-terminal juxtamembrane region of APPTM (Figure 3A) targeting specifically the juxtamembrane triple Lys cluster K53-K55. APPTM is shown to have an MW increase by MALDI-TOF-MS, which indicates that 6H8 covalently binds to APPTM. Combining the results of NMR and MALDI-TOF-MS, it is apparent that 6H8 covalent modifies at the C-terminal Lys cluster of APPTM. This location of covalent modification is advantageous as it is near the initial ε-cleavage sites of presenilin, T48, and L49 (Takami et al., 2009). Through mutagenesis, the juxtamembrane residues of APPTM have been shown to play an important role in GS cleavage (Kukar et al., 2011; Li et al., 2017; Yan et al., 2017). Our previous NMR studies established that the C-terminal lysine cluster of APPTM undergoes the largest CSPs upon initial docking of PSH, revealing their importance during intramembrane proteolysis (Clemente et al., 2018). Upon docking to PSH, APPTM exhibits a pattern of reduced chemical shifts of amide protons at the C-terminal half of APPTM indicating weakened helical hydrogen bonds, due to the unwinding of the α-helical geometry (Clemente et al., 2018). These studies reinforce the C-terminal region of APPTM as a promising drug target for novel drug discovery targeting inhibition of APP by GS.

In our gel-based cleavage assay using PSH, 6H8 modified APPTM significantly inhibited cleavage of APPTM/APP by PSH with an IC_50_ range between 2 and 4 μM (Figure 4). Inhibition of GS cleavage of 6H8 modified APPTM can be rationalized by the cryo-EM structure of the APP C83/GS complex. In this complex APPTM takes on an extended *β* conformation that exposes the ε-cleavage sites by forming a β-sheet with two β-strands from PS (Marambaud et al., 2003). 6H8’s modification of APPTM at the C-terminal Lys (K53-55) likely interferes with the *α* to *β* transition and/or formation of the β-sheet complex between APP and presenilin, thus inhibiting cleavage by presenilin and ultimately GS.

One major concern with covalent modifying drugs is the potential for off-target reactivity and promiscuous binding. While conventionally the pharmaceutical industry avoids covalently modifying drugs, many first-in-class drugs like aspirin and penicillin covalently modify their targets and encompass roughly 30% of the current drug market (Roth et al., 1975; Wright et al., 2014). Despite the traditional hesitation toward covalent drugs, there are considerable benefits to these drugs, such as a zero-off rate and high efficacy. Both factors facilitate lower concentration and less frequent dosing, which assists in mitigating off-target and side effects while dramatically increasing patient compliance (Bauer, 2015). There has been a surge in covalent drug candidates in development specifically targeting “undruggable” targets, like APPTM, that have no obvious binding pocket. While 6H8 is a small fragment that is potentially promiscuous, we plan to design a targeted covalent inhibitor (TCI) using 6H8 that combines the benefits of covalent and non-covalent inhibitors (Singh et al., 2011). Our plans for TCI development include using 6H8s as the covalent warhead attached to a non-covalent binding linker.

Inhibition of the active subunit of GS (presenilin) limits the production of Aβ and overall senile plaque load in the AD brain. Here we report the discovery of 6H8 a fragment that covalently modifies APPTM to inhibit cleavage by PSH and by extension GS. This study serves as a follow-up to our lab’s initial discovery of novel covalent modifier C1, in which we demonstrated that targeting the substrate of GS alone can sufficiently reduce Aβ production (Barthet et al., 2013). This method of targeting the substrate of GS, APPTM, continues this new direction in AD discovery targeting amyloid load as the disease-modifying strategy. This methodology can readily be applied to other “non-druggable” targets where there is a vested interest in maintaining the normal function of the enzyme that cleaves other physiological substrates.

## Summary/conclusion

### Targeting the substrate of GS (APP) allows for normal function of GS while reducing the generation of amyloid plaques

One major pathogenic hallmark of AD is the formation of senile plaques (Price et al., 1998). These plaques are formed *via* intramembrane membrane proteolysis of APP by GS. Given its role in plaque formation, GS inhibition has been a traditional target for AD drug discovery, with limited success. The 90 + endogenous substrates of GS, and two notable failures of broad-spectrum GSIs (avagacestat and semagacestat) in clinical trials, suggest that eliminating GS function is not the best approach to reducing the overall amyloid load (Coric et al., 2012; Doody et al., 2013; Coric et al., 2015; Ferrero et al., 2016; Abyadeh et al., 2021). We hypothesize that specifically targeting APP to reduce amyloid load while retaining the necessary functions of GS.

### NMR screening of the maybridge library yielded fragment 6H8 which serves as an inhibitor of intramembrane proteolysis of APPTM by presenilin homologue

Initial screening using APPTM (transmembrane domain of APP) as the target produced 6H8 as a binding fragment (representative spectrum Figure 3A). Binding events to APPTM are observed through selective line broadening at or near residues interacting with 6H8. Using 2D TROSY NMR we determined that 6H8 binds at the C-terminal triple lysine cluster K53-K55 (Figures 3A,C). Inhibition efficacy of 6H8 was determined using a PSH gel-based cleavage assay where PSH is an archaeal homolog of the active subunit of GS (Figure 4). IC_50_ determined by this assay was between 2–4 μM, putting it in a promising range for future drug development. (Hughes et al., 2011).

### 6H8 covalently binds to lysine sidechains transmembrane domain of amyloid precursor protein *via* a michael addition after initial oxidation from hydroquinone to active quinone form

After observing a color change in the stock solution of 6H8, we determined the compound was autoxidizing in our aqueous experimental buffer (25 mM Na_2_PO_4_, 4% DPC, pH 7.2). Based on this observation, we performed NMR binding studies and PSH cleavage assays in the same reaction conditions with the addition of the reducing agent TCEP (1 mM for NMR studies, and 1 mM for PSH assays). The ability of 6H8 to interact with APPTM and inhibit PSH cleavage was completely diminished with the introduction of TCEP (Figure 6).

Given the simplicity of the fragment’s structure, there are limited oxidation sites, with the hydroquinone moiety being the most likely location, as the moiety is readily oxidized to a quinone. The quinone moiety provides an *α*, *β* unsaturated ketone (Michael acceptor) to the amine group at the end of the Lys sidechain (Michael donor). The Lys amine performs a nucleophilic attack on the unsaturated ketone of 6H8 following a standard Michael addition mechanism (Figure 7) (Radel et al., 1982; Yadav et al., 2008). The covalent modification was confirmed *via* MALDI-TOF-MS where up to six additions of 6H8 to APPTM can be observed after 16 h incubation at 37°C (Figure 5).

### Covalent fragments can serve as a reactive covalent warhead in rational drug design of targeted covalent inhibitors

While a covalently modifying fragment is not ready for implementation as a drug lead given a high probability of binding promiscuity, we propose can serve as a covalent warhead for TCI development. TCIs offer the specificity of a non-covalent binder with the zero off rate of a covalent inhibitor. (Singh et al., 2011; Wright et al., 2014; Bauer, 2015). Based on NMR titration data, 6H8 binds to the transmembrane region of APPTM and inhibits cleavage by PSH, and is readily available for addition to a non-covalent binder of APPTM.

## Materials and methods

### Transmembrane domain of amyloid precursor protein overexpression and purification

The fusion constructs maltose-binding protein APPTM (MBP-APPTM) was transformed into BL21DE3 Codon Plus RIPL cells. Colonies with ampicillin resistance were inoculated into a 200 ml LB culture and grown O/N at 37°C. The culture was spun down, washed to remove lingering LB, and transferred to ^15^N labeled minimal media (1 M MgSO_4_, 1 M CaCl_2_, 20% (w/v) glucose, 1 g/L^15^NH_4_Cl). The culture was grown at 37°C until an OD_600_ of ∼0.6, then induced with 2 mM isopropyl β-D-1-thiogalactopyranoside (IPTG). Cultures were allowed to overexpress at 16°C for 27 h, harvested by centrifugation, and stored in −80°C until lysis.

Cells were resuspended in aqueous buffer (20 mM Tris, 500 mM NaCl, 20 mM Imidazole, 0.5 mM PMSF) for lysis *via* microfluidizer at 80 psi. Insoluble cell debris was pelleted by centrifugation (10,000 × g, 4°C), and membranes were harvested from the resulting supernatant by ultra-centrifugation using a Beckman Proteomelab XL-I ultracentrifuge (Ti45 fixed angle rotor, 40,000 rpm, 16–20 h).

Membrane pellets were manually homogenized in a 55 ml tissue grinding chamber with a smooth pestle (Wheaton 358054) using 30 ml solubilization buffer (20 mM Tris, 500 mM NaCl, 20 mM Imidazole, and 2% w/v n-dodecylphosphocholine). The membrane mixture was passed through two 5 ml Ni-NTA columns to purify His-tagged MBP-APPTM**.** Ni-NTA columns were preequilibrated with 50 ml of H_2_O and 50 ml of HisTrap Buffer A (20 mM Tris, 500 mM NaCl, 20 mM Imidazole, and 0.1% w/v DPC). Solubilized membranes were loaded onto the column, washed with 100 ml HisTrap A until A_280_∼0, and eluted with an isocratic elution of HisTrap Buffer B (20 mM Tris, 300 mM, 250 mM Imidazole, and 0.15% w/v DPC).

Before cleavage by thrombin, the protein was dialyzed into thrombin digest buffer (10 mM Na_2_HPO_4_, 140 mM NaCl, 3 mM β-mercaptoethanol, 0.05% w/v DPC). MBP-APPTM was digested by 10 U of thrombin/mg of fusion protein at room temperature for 48 h. Post cleavage, APPTM was separated from the cleaved tag using Ni-NTA following the same steps as previously described. APPTM was dialyzed into NMR buffer (25 mM Na_2_PO_4_ pH 7.2) and concentrated up to 4% DPC. Protein concentration determined by BCA assay.

### NMR-based fragment library screening

Using APPTM as the target fragments of compounds from the Maybridge Ro3 1000 library (under 200 Da) were screened *via* NMR spectroscopy. Positive hits were marked by significant chemical shift perturbations (CSPs) and/or selective line broadening on a ^1^H-^15^N 2D transverse relaxation optimized spectroscopy (TROSY) NMR spectrum of APPTM. Negative hits result in a TROSY spectrum identical to the APO APPTM spectrum. Multiple positive hits were obtained using this screening technique, including C1, the covalent modifier of APPTM which we previously published (Barthet et al., 2013). The library was screened by pooling ten compounds and incubating them with APPTM at 318 K. Samples that yielded positive results were parsed by individually incubating the compounds with APPTM allowing for the identification of individual positive hit compounds.

### Gel-based cleavage assay by presenilin homologue MAMRE50 was used to determine efficacy inhibiting intramembrane proteolysis

A gel-based assay previously established in this lab to monitor the cleavage of APPTM and 6H8 modified APPTM by presenilin homolog MAMRE50 (PSH) was utilized (Zhao et al., 2020). APPTM (5 μM) was incubated with varying concentrations of 6H8. Native APPTM and 6H8-modified APPTM were incubated with PSH (20 μM, 37°C, 24 h) before running on a 12% SDS-PAGE gel (200 V for 35 min). III-31-C, a known γ-secretase inhibitor, was pre-incubated with PSH for 1 h before adding the substrate serves as a positive control for complete inhibition. Initial IC_50_ PSH assay utilized a range of concentrations of 6H8 from 0.2 to 20 μM (0.2, 0.4, 0.6, 0.8, 1, 5, 10, 15, and 20 μM) (Figure 4A). Following initial PSH assay another assay was performed using a smaller range to narrower the IC_50_ range (1, 2, 4, 6, 8, and 10 μM).

### Solution NMR

Original ^15^N-^1^H TROSY spectra of APPTM *via* solution NMR has already been accomplished and reported in the BMRB (Entry 18649) (Zhao et al., 2020). A well-resolved ^1^H-^15^N TROSY spectra were recorded in 10% D_2_O on an 800 MHz Bruker Advance II spectrometer equipped with cryogenic probes. Titrations of APPTM with 6H8 were monitored for both chemical shift perturbations (CSPs) and selective line broadening. CSPs are indicated by a linear shift and are indicative of fast exchange in the system, whereas selective line broadening corresponds to intermediate exchange in the system leading to a decrease in peak height of affected residues. 6H8 was added to a ^15^N-labeled APPTM sample at a ratio of 1:10. ^1^H-^15^N TROSY spectra were collected for APPTM before and after adding 6H8 at 318K. Spectra were analyzed using Sparky. (Lee et al., 2015).

### MALDI-TOF-MS

MALDI-TOF-MS was applied for the detection of 6H8 covalent modification of APPTM. All reactions were performed using ^15^N labeled APPTM in NMR buffer. Detergent in all samples was removed (Pierce™ Detergent Removal Spin Columns 87777) for better detection. MALDI-TOF-MS spectra of APPTM were acquired on a Bruker Daltonics-autoflex™ speed MALDI-TOF/TOF spectrometer. A linear mode was applied for detecting APPTM with sinapinic acid as the matrix. MALDI-TOF-MS spectra of APPTM (5,590 Da, 50 μM) with a 1:5 ratio of 6H8 treatment after 16 h incubation at 37°C.

## Data Availability

The raw data supporting the conclusion of this article will be made available by the authors, without undue reservation.
